# Book Reviews

**Published:** 1813-01

**Authors:** 


*Edinburgh Medical and Surgical Journal, No.* XXXI.

(Continued from Vol. 28, p. 512.)

VIII. *Observations on the Cause of Death from what is called the Wind of a Ball; by the* Rev. PATRICK FORBES.—Mr. Forbes is dissatisfied with the explanation of this phenomenon by Mr. Ellis, who refers it to some modification and peculiar action of the electric fluid; and also to the opinion of Dr. Spence, who, he says, " seems inclined to believe that the accidents in question are owing to the velocity communicated to light substances by the ball." Having shortly examined and rejected the opinions of Mr. Ellis and Dr. Spence, Mr. Forbes presents an hypothesis of his own.

" I think (he says) that there is a mechanical cause for the effects produced by a ball passing close to the human body, which will sufficiently account for them. This is the vacuum produced by the rapid motion of the ball through the air, which, though of itself partial, yet when we combine with it the condensation of air taking place immediately before the ball, may, I suppose, have fully the same effect as if the vacuum behind it were complete. When a ball then passes close to the stomach, there is, in the first place, a great addition to the pressure on that viscus from the condensation of the air; as soon as the ball is passed, this pressure, with a great part of that of the atmosphere, is taken off; the consequence of which is a sudden expansion of all the fluids in the stomach, and the blood in its blood-vessels, and the rupture of both. The rupture of the stomach is the cause of death, and the extravasation of the blood, of the black appearance, externally. In the case mentioned of the false-ribs being fractured and death following, the same explication will apply; some viscus, from sudden expansion of its contained fluids, has burst and forced the ribs outwards. The case of the thigh-bone being broken, and about two inches of it being shivered into small pieces, appears a great confirmation of this mode of explaining the effects produced, and is very hostile to that of Mr. Ellis. Had the accident been occasioned by electricity, the thigh ought to have been shivered lengthwise by the passage of the fluid to the earth. But, if occasioned by a vacuum suddenly produced on the one side, and by the whole pressure of the atmosphere immediately applied on the other, this will account for the two inches being, as it were, broken out from the femur; and the shivering into small pieces was caused by the sudden expansion of the fluids in the internal and vascular part of the bone."

In the former numbers of this Journal, the opinions of Mr. Ellis and Dr. Spence are stated with sufficient explicitness to enable our readers to form a judgment upon them. We still think the subject not devoid of interest, though yet obscure.

IX. *Observations on the Treatment of Chorea Sancti Viti; by* HENRY REEVE, M.D.—The various and opposite methods of treating *Chorea Sancti Viti*, is the subject of Dr. Reeve's paper; and he was induced to select this disease for a comparative statement, "because in it two of the three points of evidence necessary for establishing medical facts can be readily ascertained. viz. first, that the disease really did exist; and, secondly, that it was cured. The third point,—whether the alleged means alone performed the cure, is a question which may be left for the reader to determine."

The following report of the occurrence and progress of this disease in a public hospital for thirty-six years, under the management of eight different physicians, we consider to be a document of value, because it presents facts in the history of Chorea.

"*A General Abstract of the Patients with Chorea, admitted and discharged from the Norfolk and Norwich Hospital, from March* 1776, *to March* 1812.

| | | No. |
|---|---|---|
| Patients affected with chorea, admitted from the month of March 1776, to March 1812 | | 84 |
| Discharged | Cured, | 74 |
| | Relieved, | 5 |
| | At his own request, | 1 |
| | Having epileptic fits, | 1 |
| | Not likely to receive benefit, | 1 |
| | Died, | 2 |
| | | 84 |

"It appears that this disease occurred in 84 out of 28,810 patients, (the total number admitted,) or 1 in 343. It is most frequent in females, in the proportion of 57 to 27, or as 19 to 9. Out of the number of cases above stated, there was only one man, forty years of age, and that was a severe case; for the disorder returned a few months after he was discharged from the hospital; he was re-admitted, admitted, and continued eight months under medical treatment till he was dismissed relieved. All the patients except two girls, aged five and six, were above nine years old; the most common date of the commencement of the disorder, was between the ninth and fifteenth year of the patient's age; four females were above the age of twenty-one. Out of 84 cases, only two had a fatal termination; both of these were boys, in whom the disease had been of long standing. The patient discharged as not likely to receive benefit, was a woman twenty-eight years old, who had been affected at different times for three years prior to her admission; the patient discharged at his own request was a boy, who had the disease a year before he came to the hospital; he remained there four months. The patients relieved were persons who had been long affected; and the one discharged on account of his being subject to epileptic fits, was a lad fifteen years old.

"Relapses took place in nine cases, all of them after an interval of not less than a year; in three of these the disease recurred a third time, but was ultimately cured. The shortest period of medical treatment was two weeks; the longest, eight months; the common average, seven weeks; observed in the whole 84 cases."

Dr. Reeve has seen, in the course of sixteen years, thirty-five cases of chorea. All these patients recovered except one, and they got well under every variety of treatment, from the most powerful down to the most inert and apparently inefficacious remedies. The fatal case was that of a boy twelve years old, in St. Bartholomew's Hospital, in 1804. This patient took scammony and calomel in large and repeated doses, besides other medicines, without any abatement of the symptoms; which were convulsive motions, violent and continual, night and day, for six weeks, when he died exhausted.

Dr. Reeve admits that the evidence in favor of purgatives in the cure of chorea is very decisive; and does not doubt but this method of treating the disease is the speediest and the most effectual; but he argues that it is not proved that tonics and stimulants have always failed, and that purgatives alone will be universally successful. Instead of tonics and stimulants always failing, our author states, that the late Dr. Lubbock, of Norwich, always prescribed preparations of steel for the cure of chorea; he never employed purgatives or evacuants of any kind, and he never failed of success.

It is evident that the same mode of treatment will not suit every case of chorea, nor the same case at all periods of its progress. The alternation of tonics and evacuants, and the keeping up the strength by nutritious and stimulant food, may often be highly proper. After all, the third point, which Dr. Reeve leaves to the reader's determination, will long remain doubtful. In a disease, the essence of which is a disordered disordered condition, and inordinate action of the functions of the nerves, much must be referred to spontaneous changes, or allowed to accidental events. The habitual morbid action of the nerves may receive some unobserved impression, equal to break the diseased concatination, while a favorite remedy is exhibiting, which necessarily receives the credit of the cure. Dr. Reeve saw cases of chorea cured by inert remedies, which he, singularly enough, denominates also inefficacious, and many obstinate and apparently irremediable epilepsies have been removed by strong mental impressions.

---

*A Manual of the Treatment of Parturient Animals. By* T. Parkinson, M.D. 12mo. Nottingham. 1812. pp. 8.

This short synoptical view of the symptoms and treatment of a state in animals of the first importance, we consider as conveying so much useful information disposed in such a perspicuous manner, that we cannot do the author greater justice, and the public more benefit, than by repeating it nearly verbatim, in the form in which he has presented it to us.

To convey useful knowledge in a short, clear, and cheap, form, is no slight excellence; and this excellence Dr. Parkinson has attained in his *Manual of Parturition.*

"*Antecedent Signs of approaching Parturition.*

| SYMPTOMS. | TREATMENT. |
|---|---|
| Restlessness; solitude; labia enlarged; mucous discharge from the vagina, without forcing pains. | Nothing. |
| *Commencement of Parturition.* | |
| As above, with pains at short intervals; appearance of membranous bag of waters. | Examination, to ascertain the nature of the part of the young presenting; the degree of dilitation of the os uteri. |
| Pains increasing in strength and forcing downwards. The os uteri dilated to the size of half-a-crown. | The presentation fully ascertained to be natural; nothing. |
| *Art of delivering Animals of their Young.* | |
| | The whole attention to be directed to the state of the os uteri, which is to be kept acquainted with by frequent examinations; but done with all care and tenderness. |
| *Examination per Vaginam.* | |
| The nose and the two fore feet feet presenting, the feet a little before the nose. | Patience, till the os uteri is fully dilated, which can then no longer be felt by examination. |

Natural

*Natural Presentation.*

| | |
|---|---|
| The nose proceeding through the external orifice, the feet not proceeding so fast as the nose. | Nothing. |
| The pains strong, without any progress. | Gentle traction, at the lower jaw and the feet, but not at the feet, or at one foot, without corresponding force at the jaw. |

*Practice in Natural Presentations.*

| | |
|---|---|
| The os uteri fully dilate; but no progress, and the pains feeble or ceasing. | As above. |
| The head born, the feet proceeding but little. | Employ the extracting force at the head only; but not at the feet, that the shoulders may be more conveniently born, by the elbows remaining somewhat behind the shoulders. |
| The head and shoulders born. | Employ some force at the feet, and speedily finish delivery. |
| The nose presenting without the feet, the os uteri fully dilated. | Put back the nose and obtain the feet; but do not bring the feet too forward; then proceed as above. |
| The nose and only one foot presenting. | Put back the nose, and obtain the other foot; then proceed as above. |

*Practice in Preternatural Presentations.*

| | |
|---|---|
| Both feet without the nose presenting. | Put back the feet and adjust the head; then proceed as above. |
| One foot only presenting. | Obtain the other foot; then put both feet back, and adjust the head; then proceed as above. |
| Both the hinder feet presenting. | Patience till the breech is born; then finish delivery with all expedition. |
| One hinder foot only presenting. | Disregard the other foot; have patience till the breech is born, then deliver with expedition. |
| The breech or tail presenting. | Have patience till the breech is born; then hook the fore fingers in the groins, and deliver with expedition. |
| The nose presenting with the face downwards, or towards the pubis. | Turn the face towards the sacrum; then proceed as in a natural presentation. |
| Plurality of young. | Proceed with each according to their respective presentations. |

*Management of the Placenta.*

| | |
|---|---|
| The young being born. | Patience after tying the funis umbilicalis, and separating from the mother. |
| Retained placenta. | Introduce the hand into the uterus beyond the placenta; if the uterus be contracted, and the placenta is concealed beyond the place of contraction, such contraction to be dilated, and the hand then carried on till it has passed to the furthest part of the placenta, which is to be brought away entire before the hand; protect against severe cold. |

MEDICAL TREATMENT.

Give the mother her natural food only, and water a little warmed.

| | |
|---|---|
| Pains continuing after the process of delivery is fully accomplished, with heat and a white tongue. | Bleed from a vein; give an opiate, and small quantities of nitre in the water, and, if needful, magnesia vitriolata as a purge; a warm bran mash with a few oats in it. |
| Strength returning. | Return to natural food, to cold water, and the fields. |

---

*An Account of a Case of Recovery, after an extraordinary Accident, by which the Shaft of a Chaise had been forced through the Thorax. By* WILLIAM MAIDEN, Surgeon. 4to. Lond. 1812. pp. 28.—*Bayley.*

THE extraordinary accident narrated in this pamphlet, happened on the 13th of June last, to a gentleman named *Tipple,* a resident in Hoxton, a village in the vicinity of London. Having returned from town on the above day, in a single-horse chaise, to a place near Stratford, Mr. Tipple inadvertently removed the bridle from the horse's head before the animal was liberated from the carriage, when, immediately plunging forward, he forced the shaft of the chaise between Mr. Tipple's ribs on the left side, into the cavity of the thorax, under the sternum, out between the ribs on the right side, and then through the weather-boarding of the chaise-house; so that the patient was literally impaled, and fixed to the building.

Mr. Maiden, a surgeon of Stratford, saw him soon after the accident, and furnishes the following narrative.

"I found Mr. Tipple in bed, supported by several pillows; and, his left side being towards me, I proceeded to examine him, when, to my great surprise, I perceived air with blood, issuing freely from a wound, which I afterwards found to be the lower wound, inflicted by the iron under the shaft.

" Observing him to breathe with extreme difficulty, I advised him to keep still, and to answer my questions with as little exertion as possible. I then asked him, whether he felt pain in his back ? He answered, ' No; what I feel is a dreadful weight on my chest, as if I should be suffocated by the blood trickling on my lungs'. Being apprehensive he was suffocating from inward hemorrhage, I opened a vein in his right arm with a very large orifice.

" Finding the oppression gradually relieved, and that the pulse supported the evacuation, it was allowed to proceed till at least four pounds (avoirdupois) of blood were taken, when fainting came on; upon which the attendants were eager to apply hartshorn, &c. but I advised them to desist, allowing them only to give him a little cold water.

" Nothing more was done until Sir William Blizard arrived, which was at about half-past eleven. We proceeded to examine the lower wound on the left side, supposing that to be the part where the shaft had entered, when Mr. Tipple himself informed us that ' this was the wound made by the iron under the shaft; that the wound where the shaft entered was higher up, immediately under the arm;' and, he added, that ' there was also a wound on the right side, nearly in the same direction, through which the shaft came out.'

" On examination we found a corresponding wound under each arm, not less than four inches each in extent.

" The left shoulder and side of the chest were, in a small degree, emphysematous.

" Sir William expressed his opinion, that Mr. Tipple would not live over the night, observing, that, as soon as the action of the heart revived, the internal hemorrhage, which had been checked by the copious bleeding, would return, when he would sink. We left him between one and two in the morning, under this impression.

" During the time I was waiting for Sir William, I examined the shaft of the chaise very minutely, and observed that it was covered with blood, as far up as where it was supported by the tug; some small pieces of fleshy substance sticking to the tip of the front tug-hook, where it was turned backwards, and a piece of flesh, about the length of the little finger, hanging to a splinter of the shaft, between three and four inches from the tip.

" Sunday, the 14th of June, at eight in the morning, I again visited Mr. Tipple, and, to my surprise, found him nearly in the same state in which I had left him; and was informed that he had enjoyed some sleep. I directed the greatest attention to his quiet; and that nothing more than a little cold water should be given to him, as I was still apprehensive that the internal hemorrhage would return.

" Sir William Blizard, being again sent for, visited him about the middle of the day; and advised a draught of the infusion of roses, with a drachm of the sulphate of magnesia, every sixth hour.

" We met on Monday the 15th, at eight o'clock a. m. and, finding the the difficulty of breathing much increased, with considerable pain, weight, and soreness, in the chest, he was again bled to the amount of thirty ounces, and became much relieved.

"The draughts having operated but slightly, and Mr. Tipple complaining of great fullness about tne stomach and bowels, with some degree of nausea, an enema with castor-oil, &c. was administered in the evening, and five grains of calomel were ordered to be taken at bed-time.

"Tuesday, June the 16th, the calomel had operated gently two or three times, but the patient had been very restless, and had vomited frequently. He complained of considerable pain about the region of the diaphragm, attended with the same difficulty of breathing, and soreness in the chest, as described the morning before; whereupon eighteen ounces of blood were taken from the arm, which gave immediate relief. Glysters were repeated; and, in the afternoon, finding that the vomiting had increased, with hickuping also, the saline draught, in the state of effervescence, was prescribed, which happily relieved those very distressing symptoms.

"The fatal termination, which had been hourly expected from the beginning of the accident, seemed, when I visited him this night, fast approaching.

"But on Wednesday, June the 17th, we found he had passed a better night, with some sleep: the difficulty of breathing being, however, greatly increased, with the soreness of the chest, seventeen ounces of blood were taken away, in consequence of which these symptoms were alleviated. The calomel, which had been repeated each night, having acted freely, his stomach and bowels were also much relieved.

"As Mr. Tipple's position was seldom altered for the first fortnight, without my assistance, and this extraordinary accident weighing constantly on my mind, I took occasion to ask him, from time to time, while assisting him with my hand under his back, whether the pressure gave him any pain? and he always replied, it did not.

"This day, in the afternoon, while an assistant and myself, with our hands under his back, were gently raising him on his pillows, I particularly inquired, whether he felt any pain or soreness in his back? when he assured me he did not; and, doubtful whether any rib were fractured, I questioned him at the time of dressing his wounds, if he felt any pain in either side? he told me, no other than the smarting of his wounds, observing, as he had done before, that he thought, from the pain and great tenderness about the breast-bone, that it was broken.

"Thursday, June the 18th.—This morning we found his breathing very laborious, whereupon twenty-two ounces of blood were drawn; and, notwithstanding he was much relieved in point of respiration by this measure, he still complained of general tenderness in the chest and epigastric region.

"Although the calomel had acted rather freely, it was deemed adviseable to add to its effect, by occasional doses of the sulphate of magnesia with the infusion of senna.

"The symptoms in the morning having appeared urgent, in the course course of the day I received a letter from Sir William Blizard, advising me if I conceived it to be necessary, to apply a very large blistering plaster over the sternum.

"Friday, June the 19th.—This day the effects of the blistering plaster, and the very free evacuation of the bowels, rendered the repetition of bleeding unnecessary, but it was thought adviseable to continue the aperient.

"Saturday, June the 20th.—This morning the symptoms were not so urgent as to induce us to repeat the bleeding; but, in the evening, respiration becoming more difficult, nineteen ounces of blood were taken away.

"This day I observed several threads of flannel, deep in the wound under the right arm, which must have been rent from the under waistcoat, by the splinter near the end of the shaft as it was withdrawn.

"Sunday, June the 21st.—The symptoms being moderated, the treatment was similar to that of Friday; and broth was added to his diet, which, to that period, had been only of vegetable matter.

"Monday, June the 22d.—This morning less pain and difficulty of breathing than since the accident; but the patient had distressing sensations about his chest, which, he said, he could not well describe; and, the pulse admitting the measure, he was again bled.

"Now, for the first time since the accident, Mr. Tipple, while supported, sat up in bed, and we were enabled to ascertain the situation of the wounds, and especially to examine the back, in no part of which the smallest trace of injury could be perceived; on the contrary, the integuments over the spine and shoulders appeared perfectly healthy and pliable, without swelling, or discoloration, or the least pain or tenderness upon being pressed.

"As soon, therefore, as Mr. Tipple was recovered from the fatigue of changing his shirt, &c. I took away fourteen ounces of blood, by which he was more relieved than he had been before, not feeling any pain, only a smarting sensation, similar to that which he experienced in the wounds under his arms, on each side the breast-bone, internally, in the direction in which he was convinced the shaft had passed.

"As the blistered surface, from the plaster on Thursday, was nearly healed, another, larger, was directed to be applied; and the bowels to be frequently moved. By these means the symptoms were so much abated as to render any further recurrence to the lancet unnecessary; and on Thursday the 25th of June, the alarming effects of the injury had so far subsided, as to admit of a reasonable hope of Mr. Tipple's recovery. Nevertheless, as his situation remained critical, the blistered part was kept freely discharging; and the aperient medicines were frequently administered for several days. He continued gradually to recover, and the wounds to heal, which, from the extensiveness of the laceration, were, however, scarcely closed at the end of nine weeks."

From the evidence furnished by Mr. Maiden, from the opinion of Sir William Blizard, and from the admission of many many gentlemen who saw the case during the progress of the cure, or since the patient's recovery, a doubt can hardly remain of the shaft of the chaise having passed through the cavity of the thorax, from the left to the right side. The favorable termination of this extraordinary wound, distinctly shews what enormous lesions of parts considered essential to the existence of the animal machine, may be endured without the loss of life, under judicious management. Two points, in this instance, were to be considered as those on which the life of the patient was suspended—one immediate, the occurrence of fatal hæmorrhage—the other remote, the consequence of inflammation. By evacuations carried to great extent, and by rigid discipline as to nutriment, both the dangers were obviated. To the judicious application of the lancet, principally, the patient probably owes the preservation of his life. At the first bleeding, immediately after the accident, sixty-four ounces, and within the first eight days, 186 ounces of blood were abstracted. In the first period, the patient was kept on water alone; and, in the progress of the case, resort was had to the auxiliary aid of cathartics and blistering.

---

*An Account of the Ravages committed in Ceylon by the Small-pox, previously to the introduction of Vaccination; with the Statement of the circumstances attending the introduction, progress, and success, of Vaccine Inoculation in that Island. By* THOMAS CHRISTIE, M.D. 8vo. Cheltenham, 1811. pp. 104.

THE extension of the practice of vaccination to the remote parts of the globe, among the wandering nations of the American continent, among the vast population of China and Hindoostan, the barbarous hordes of northern Asia and of Africa, to both the polished and the savage islands of the Pacific Ocean, among people of all religions and of all opinions, speaks most powerfully in justification of its utility. In no place and in no instance, however, has its success been more obvious and unalloyed than in the island of Ceylon. In this island, marked by heaven to suffer by one of its severest scourges, the devastation occasioned by small-pox presents a picture of peculiar horror. By the wide wasting variolous pestilence, towns and villages were depopulated; dismay accompanied its steps, and destruction closely followed its progress. The state of Ceylon, under the affliction of the casual small-pox; the attempt made to stem the torrent of desolation produced by this disease, by the introduction of small-pox inoculation; and the final employment of vaccination, to the the entire annihilation of the variolous contagion; are the points treated in Dr. Christie's publication.

The sufferings of the Cingalese by variola afford a picture of human calamity, which, possibly, has never occurred in Europe.

"On my first arrival in Ceylon, (says Dr. Christie) in 1797, when it was under the administration of the East-India Company, I was too little acquainted with the language or manners of the country, and too much occupied by the duties of a sickly regiment to which I was then surgeon, to have acquired much information about the progress of small-pox, or other diseases, amongst the natives, even had I been in a more favorable situation for such enquiries, than Trincomalie, the country round which was then but thinly inhabited.

"My attention was not, in consequence, directed to this subject, till the year 1799, when small-pox appeared in the Pettah, or Black town of Trincomalie; soon after which, a case occurred in the Fort, in the person of an European woman belonging to the 80th regiment, who was near the time of her delivery. This patient, Margaret Carnie, sickened on the 10th of November, 1799, was delivered on the 12th, and died on the 17th, of confluent small-pox, the contagion of which was evidently traced from a native, affected with the disease. Her infant, which was born in apparent health, died of convulsions a few days afterwards, antecedent to the appearance of any eruption.

"These circumstances were detailed by me in a letter to the late Dr. John Ewart, then physician-general in Ceylon, on the 18th of November; when, with the consent of the commanding officer of the garrison, seventeen men, and a proportion of European children and natives attached to his Majesty's 80th regiment, were received into hospital, and inoculated, all of whom did well.

"A proclamation was at the same time issued by the magistrate of the place, exhorting the inhabitants to adopt inoculation, and offering my gratuitous services and attendance, but, although at the time numbers were daily dying from natural small-pox, not a single inhabitant would submit to the operation, except the native followers of the 80th regiment formerly mentioned, and a few gun lascars, who were inoculated by Mr. Rogers, assistant-surgeon to the Madras Artillery. Indeed, such was the prejudice of the natives against inoculation, or any interference with what they considered as a visitation of their gods, that it was with the greatest difficulty permission could be procured to take a little matter from a child, for the purpose of inoculating the Europeans.

"This, agreeably to the best information I have been enabled to procure, was the first trial of inoculation in Ceylon, since the capture of the island by the English; and, although the number inoculated at this time did not exceed fifty or sixty, yet, as all these cases were successful, it paved the way for its more general adoption, and for the establishment of the Small-pox Hospitals, the first of which was opened at Trincomalie, early in the following year, under my immediate superintendence.

"The small-pox continued to make considerable ravages during

1799 and 1800, at Trincomalie, and still more so in the other parts of Ceylon, particularly in the south-west side of the island, where it occasioned so great a mortality as to be considered in the light of a pestilence, in consequence of which the natives deserted their villages and infected relatives, on its first appearance, to the great diminution of the revenue, and the infinite distress and misery of the inhabitants.

"In September, 1800, I was witness to a scene of this kind, which I cannot find words fully to describe, but shall merely attempt to give some idea of it, by copying the official report which I made at the time to the Committee for superintending Charitable Institutions in Ceylon, of which the Hon. Frederic North Govenor was patron, and General M'Dowal president.

"'The small-pox broke out suddenly, in the middle of July, i Errore and Undewally, two villages in the neighbourhood of Batticalloa, and so great was the panic occasioned amongst the inhabitants by the appearance of the infection, that all those in health immediately deserted their habitations, and left the helpless sick without any assistance.

"'I visited the village of Errore on the 4th of September, at which time the infection had ceased, and the people had begun to return to their usual habitations, but found their former residence (lately a flourishing village) almost entirely waste and desolate, in consequence of their precipitate desertion. Out of thirteen infected persons, six had died; and the others were just recovered, and in a miserable state.

"'The survivors made the following melancholy recital, which was too certainly verified by the appearance of the village. Soon after the village was deserted by the inhabitants in health; the elephants, cheetas *(panthers)*, and wild hogs, came down from the Jungle, broke down the fences, destroyed and rooted up the trees, ate the stores of paddy (unhusked *rice)* and other provisions; and, what is still more horrible, carried off some of the sick, or at least consumed the bodies of the deceased. It is certain, that, in one house where three sick persons had been left, not a vestige of even their remains was to be found on the return of the inhabitants.'"

Another writer, *Cordiner*, (Description of Ceylon, vol. i. p. 253) describes this dreadful scene in a manner that illustrates and confirms the preceding narrative.

"On the 4th of September," Cordiner says, "I accompanied the superintendant of hospitals, in a boat, ten miles up the arm of the sea which beautifies this part of the country. His object was to inspect the villages of Vandermal and Eraoor, which had been lately deserted by the inhabitants, owing to the breaking out of the small-pox. On such occasions the husband forsakes his wife, the mother her children, and the son his father, often leaving them in their miserable huts to the ravages of famine and wild beasts of the forest. Sometimes, however, they contrive to furnish them with subsistence without entering their dwellings; but, in those sequestered hamlets, medical aid was only now beginning to be known. For many years great desolation was committed annually by that exterminating malady the natural small-pox. The Dutch inhabitants, being themselves enemies to the

 practice practice of inoculation, never encouraged it among the native Cey-lonese.

" After the inhabitants had left their villages, they became the prey of wild elephants, hogs, and cheetas. The elephants broke down the feeble fences, took possession of the gardens, tore up the plantain trees by the roots, levelled those of the cocoa nut, and browsed on their leaves. The ravaged orchards exhibited scenes of terrible devastation, the mangled trees were strewed on the ground, the straw stripped from the roofs of the cottages, the surface of the earth broken up and filled with hollows, the fences shattered, earthen pots, the simple utensils for culinary purposes, wheels, reels, looms, and all the apparatus of the weaver, lying useless and forsaken. A family of three infected persons, who had been left in one of the gardens of Erraor, were supposed to have been devoured by the cheetas, or wild bears, as a vestige of them could not be seen. Of the diseased in Vandermal, forty people died, and ten recovered. The malady having then subsided, the villagers were beginning to return, and setting about repairing their demolished hamlets."

These distresses attracted the attention, and excited the commiseration, of the Honorable Frederic North, who about that time became governor of the island. Small-pox hospitals were established, for the reception of patients under the casual disease, and for the purpose of promoting inoculation. In 1800 Dr. Christie was placed at the head of this establishment, intended to check, at least, the devastation of the variolous contagion. Though no longer useful in Ceylon, it may be gratifying to our readers, at least it will preserve a record of the talent and active benevolence of the gentlemen concerned, with somewhat more certainty than can be expected in a loose pamphlet, to copy this plan into our pages.

" *Plan of the Small-pox Establishment in Ceylon, in conformity to the minutes of Government.*

" That the British possessions in the island be divided into four principal districts, viz. Columbo, Galle, Trincomalie, and Jaffuapatam.

" That over each of those districts be named an English surgeon as superintendant, with a salary of 200 rds. per mensem, and as many assistants as may be necessary, with salaries of 150 rds. per mensem.

" That at Columbo, Galle, Trincomalie, and Jaffuapatam, there shall be established permanent hospitals for the reception of persons of every description who wish to be inoculated; and also for the admission of such patients, infected with natural small-pox, who chuse to benefit by the institution, and who, the superintendant conceives, may be moved without risk of propagating infection.

" That in each of these hospitals shall be placed an European medical person, with a salary of 90 rds. per mensem, who shall have the entire control of it, under the superintendant of the district, to whom he shall make monthly reports, and from whom he shall receive orders.

 " That

"That there also shall be appointed to each of them a creditable burgher as purveyor, when the number of admitted patients will require it, with a salary of 40 rds. per mensem, who shall provide for the persons under cure such sustenance as may be ordered by the overseer, and shall manage the economy of the establishment.

"That in each of these establishments shall be built a convenient and spacious Bungalow for the medical overseer, and another for the purveyor, who shall be allowed to receive, on profitable terms, such patients as may wish to pay for extraordinary attendance, or convenience.

"That a register shall be kept by the overseer of all who enter the establishment, of the medicines ordered, and of the number of days they remained there; and, when the cure is perfected, he will give a certificate, and receive one from them, which, being transmitted to the superintendant, and presented by him to the collector, or civil paymaster; will entitle the overseer to receive two rds. on account of government.

"The purveyor will receive, at the end of each month, pay for the expenses of the patients, at the rate of three fanams per diem, according to the register; the said register being signed by himself and the overseer, and transmitted to the superintendant, to be presented by him to the collector, or civil paymaster, who shall have orders to pay it.

"Cadjan houses shall be built for the patient, as occasion may require; and their repairs, as well as original fabrication, shall be reported to the collector by the superintendant, who shall visit them as often as possible.

"A sufficient number of nurses must be engaged, and proper attention paid to the prejudices of the people in point of cast. If the families of the patients wish to attend them, they may do so; but in that case they must submit to the regulations of the establishment, which they will not be allowed to quit, and return again to, and must live at their own expense.

"No person will be allowed to visit patients in the establishment, or to go to it, or from it, but such as are specially appointed by the overseers.

"The clothes worn by persons during their illness, must be thoroughly washed, either on their death, or on their dismissal, in sight of the overseer, who must certify the same.

"A proper guard will be given to the overseer, to enable him to enforce these regulations.

"For the benefit of persons infected with small-pox, whom it may be improper to remove into the established hospital, there shall likewise be appointed, to each of the districts of Columbo, Caltura, Galle, Matura, Batticoloe, Trincomalie, Mollativo, Jaffna, Mana, Putlam, Chilaw, and Negumbo, an European medical overseer, and such other medical assistants as may be required, who shall attend the sick at their own villages.

"Each overseer shall enjoy, at all times, a salary of 30 rds. per mensem; but, when on actual service, he shall receive at the rate of 60 rds. per mensem. The other assistants shall receive no permanent salary, salary, but, when employed in a village where small-pox prevails, they shall be allowed at the rate of 90 rds. per mensem.

"Whenever the small-pox appears in any village, the Vidaan will immediately report it the Moodiliars, who must instantly represent the circumstance to the magistrate of the district, who will, without delay, order the medical overseer, or assistant, to transport himself to the infected village, where he shall reside as long as the infection continues there.

"As soon as the overseer arrives at a village where small-pox prevails, he will undertake the regulation of the police, and will be careful, by every possible means, to prevent the spreading of infection; all egress and ingress into infected houses must be absolutely prohibited, without a passport from him; and the overseer, or ordinary magistrate, will be empowered to inflict summary punishment by the rattan, on all offenders against the regulations of the police.

"The overseer will afford to the sick every possible medical assistance which the nature of the case, or the prejudices of the people, will allow; and he will be particularly attentive to ventilation and cleanliness. The same regulations, with respect to washing the clothes of infected persons, must be observed as in the established hospitals.

"Inoculation shall not be allowed, except in the established hospitals or infected villages, where it shall be practised by the overseers, with the approbation of the superintendent; and, in that case, the families inoculated must submit to the same restrictions as in cases of natural small-pox.

"The overseers of districts, as well as those of hospitals, shall make regular reports of their proceedings to the superintendents, on whom they shall also indent for medicines.

"The abstracts for the expenses of the hospitals, and of the several overseers, must be countersigned by the superintendent, previously to their being discharged.

"Young men who wish to be employed as assistants to the overseers, must be regularly licensed, and for that purpose an adequate attendance in one of the established hospitals is absolutely required.

"The overseers of districts, when not employed, ought also to take every opportunity of visiting the hospitals for information.

"A true Extract.

(Signed) "WILLIAM BOYD,

"Acting Secretary to Government.

"*Tangalle, August* 24, 1800."

The operation and effect of this establishment is detailed. From the 1st of October, 1800, to the 30th of September, 1802, the number of patients with casual small-pox, treated by the medical overseers in the different hospitals and villages of Ceylon, was 2110, of which number 473 died, being nearly one-fourth of the whole: and the number of inoculated patients amounted to 4153, of which number 103 died, about 1 in 38. This considerable mortality occurred under the most favorable circumstances of treatment; and it is apprehended, prehended, from various evidence, that the mortality from casual small-pox among the native inhabitants, when left to themselves, was much greater. Inoculation for small-pox, by the Europeans in Ceylon, has been practised to a small extent, and not at all by the natives; so that a comparative scale of mortality by this mode of having the disease, can hardly be formed. Dr. Christie, however, conjectures, with much probability, that in a climate where the thermometer is seldom below 70°, and generally many degrees higher, that under no circumstances would the loss of life by inoculation be much below the above statement of 1 in 38. And this conjecture is strengthened by some facts relative to small-pox inoculation in India.

"The variolous inoculation by European surgeons, in most parts of India, has been chiefly confined to the children of European parents; and, from every information I have been enabled to procure, it is conceived that not less than one in forty of these died. Dr. Fleming, the distinguished head of the medical department in Bengal, has, I am told, calculated the mortality at a still higher rate, and conceives that nearly one in thirty of the children of Europeans, inoculated with small-pox at Calcutta, died.

"I am aware that it has been said, that the proportion of deaths from inoculation by the itinerant Bramins in Bengal, has been very small indeed; and, as they avail themselves of the cold season for their operations, and their patients are generally the children of natives, I can readily believe that the mortality was infinitely less than in Ceylon, where we cannot be said to have any cold season, and where a great proportion of our patients were adults; but, as the Bramin inoculators, after inserting the infected thread, or applying the infected pledgit, leave their patients to their fate, and proceed to other villages, there must be a great degree of uncertainty in their reports, and perhaps some degree of misrepresentation; and, considering the veneration in which they are held as the religious order, it is not probable, that, in the event of death ensuing, much will be said on the subject by the friends of the deceased."

(To be continued.)

